# A26 PROFILE OF ACTIVATED B-CELL EXPANSION IN THE DISTAL COLON AND MESENTERIC LYMPH NODES OF COLITIC MICE

**DOI:** 10.1093/jcag/gwae059.026

**Published:** 2025-02-10

**Authors:** M Sabzevary-Ghahfarokhi, A Marshall, J Ghia

**Affiliations:** Immunology, University of Manitoba Max Rady College of Medicine, Winnipeg, MB, Canada; Immunology, University of Manitoba Max Rady College of Medicine, Winnipeg, MB, Canada; Immunology, University of Manitoba Max Rady College of Medicine, Winnipeg, MB, Canada

## Abstract

**Background:**

Gut B cells maintain homeostasis by producing IgA antibodies that control the entry of commensal bacteria and pathogens. An increase in the number of B cells in the inflamed colon was detected in ulcerative colitis (UC) patients. Pro-inflammatory IgG antibodies binding to commensal bacteria are significantly elevated in UC, promoting intestinal inflammation.

**Aims:**

To determine the effects of intestinal inflammation on the B cell compartment using a preclinical model of UC.

**Methods:**

32 CD-1 WT males were treated for 6 days with 2.5% (w/v) dextran sulfate sodium (DSS), followed by 8 days of recovery (regular water). Disease activity index was monitored daily. Distal colonic mRNA expression of cytokine (tumor necrosis [TNF-α], B-cell activating factor [Baff]) and factors associated with B cell attraction (chemokine (C-X-C motif) ligand [CXCL]13) were assessed by qRT-PCR. Localization using B cells phenotype (B220, cluster of differentiation (CD) 19, CD138, GL7, and IgD) and activation markers (CD80 & CD86) were assessed by flow cytometry and immunofluorescence (IF) in Peyers’ patches, distal colon, and mesenteric lymph nodes (MLN). Levels of IgA, M, G1, G2b, and G2c in colon tissue and serum were determined by ELISA.

**Results:**

In colitic mice, TNF-α, Baff, and CXCL-13 mRNA expression was significantly upregulated, and immunostaining demonstrated several aggregated sites of B cells with the phenotype of B220^+^, IgD^+^, and CD19^+^in the lamina propria. Aggregated B cells were positive for co-expression of IgD and CD19 markers and negative for expression of IgA and IgG antibodies. Flow cytometry data showed a significant increase in the percentage of B220^+^ and CD19^+^ B cells within the CD45^+^ live cell population in colitic mice’s distal colon and MLNs. In addition, in colitic mice, the percentage of B cells expressing CD86 significantly increased in the distal colon, Peyers’ patches, and MLN. Furthermore, in colitic conditions, the percentage of B cells producing IgG1 was increased in the Peyers’ patches and distal part of the colon but only in Peyers’ patches for IgA-producing B cells. In colitic mice, IgA/IgG2c ratio in colon tissue decreased, and serum IgG2b, IgG2c, and IgG1 antibody levels increased, with no difference for IgA and IgM.

**Conclusions:**

Inflammatory conditions lead to B cell activation and recruitment with aggregated naïve B cells in the lamina propria that don’t switch to IgG or IgA. In colitis, systemic IgG subtypes increase, while Peyer’s patches may actively produce B cells expressing IgA or IgG1 during intestinal inflammation. These findings may guide the development of new UC therapies.

**Funding Agencies:**

CCC

